# Computer-aided evaluation of inflammatory changes over time on MRI of the spine in patients with suspected axial spondyloarthritis: a feasibility study

**DOI:** 10.1186/s12880-017-0226-4

**Published:** 2017-09-19

**Authors:** Evgeni Aizenberg, Rosaline van den Berg, Zineb Ez-Zaitouni, Désirée van der Heijde, Monique Reijnierse, Oleh Dzyubachyk, Boudewijn P.F. Lelieveldt

**Affiliations:** 1 Department of Radiology, Leiden University Medical Center, P.O. Box 9600, 2300 RC Leiden, The Netherlands; 2 Department of Rheumatology, Leiden University Medical Center, P.O. Box 9600, 2300 RC Leiden, The Netherlands; 3 Intelligent Systems Department, Delft University of Technology, Mekelweg 4, 2628 CD Delft, The Netherlands

**Keywords:** Axial Spondyloarthritis, Magnetic resonance imaging, Inflammation, Comparative visualization, Image registration

## Abstract

**Background:**

Evaluating inflammatory changes over time on MR images of the spine in patients with suspected axial Spondyloarthritis (axSpA) can be a labor-intensive task, requiring readers to manually search for and perceptually align a set of vertebrae between two scans. The purpose of this study was to assess the feasibility of computer-aided (CA) evaluation of such inflammatory changes in a framework where scans from two time points are fused into a single color-encoded image integrated into an interactive scoring tool.

**Methods:**

For 30 patients from the SPondyloArthritis Caught Early (SPACE) cohort (back pain ≥ 3 months, ≤ 2 years, onset < 45 years), baseline and follow-up MR scans acquired 9–12 months apart were fused into a single color-encoded image through locally-rigid image registration to evaluate inflammatory changes in 23 vertebral units (VUs). Scoring was performed by two expert readers on a (−2, 2) scale using an interactive scoring tool. For comparison of direction of change (increase/decrease) indicated by an existing reference, Berlin method scores ((−3, 3) scale) of the same MR scans from a different ongoing study were used. The distributions of VU-level differences between CA readers and between the CA and Berlin methods (sign of change scores) across patients were analyzed descriptively. Patient-level agreement between CA readers was assessed by intraclass correlation coefficient (ICC).

**Results:**

Five patients were excluded from evaluation due to failed vertebrae segmentation. Patient-level inter-reader agreement ICC was 0.56 (95% CI: 0.22 to 0.78). Mean VU-level inter-reader differences across 25 patients ranged (−0.04, 0.12) with SD range (0, 0.45). Across all VUs, inter-reader differences ranged (−1, 1) in 573/575 VUs (99.7%). Mean VU-level inter-method differences across patients ranged (−0.04, 0.08) with SD range (0, 0.61). Across all VUs, inter-method differences ranged (−1, 1) in 572/575 VUs (99.5%).

**Conclusions:**

Fusion of MR scans of the spine from two time points into a single color-encoded image allows for direct visualization and measurement of inflammatory changes over time in patients with suspected axSpA.

## Background

Evaluating inflammatory changes over time on magnetic resonance (MR) images of the spine in patients with suspected axial Spondyloarthritis (axSpA) can be a labor-intensive task. Depending on the rheumatologic scoring method that is used, readers are often required to assess a set of vertebral units (VUs) in several slices [1, 2], manually searching for and perceptually aligning the vertebrae between two scans. It would be of great benefit to have a computer-aided (CA) method capable of automatically localizing and labeling the VUs and spatially aligning scans from two time points, so voxel-wise intensity differences could be visualized in a single image.

CA methods involving alignment between multiple images for voxel-wise analysis have been extensively applied in the fields of neuroimaging and radiation therapy. Examples include voxel-based morphometry for comparison of local concentration of gray matter between subjects [3], analysis of multi-subject diffusion data for studying brain connectivity [4], and adaptive radiotherapy [5]. These studies have demonstrated that CA alignment of medical images can aid clinicians with automated biomarker quantification and treatment replanning based on anatomical changes that occur over time.

Spatial alignment of scans from two time points compensates for patient posture differences between scanning sessions and allows to overlay the two images for visual assessment of changes over time. This is done by computing a spatial coordinate mapping between corresponding locations in the two scans, a process known as image registration [6]. Generally, this mapping involves a geometrically non-rigid correspondence between voxels. This may cause physically implausible deformations in rigid anatomical structures, such as bones. An efficient solution to this problem was proposed by Dzyubachyk et al. [7] and applied to comparative visualization of whole-body MR scans in patients with multiple myeloma lesions. The highlight of this approach is that, following a global alignment of two time points, a locally rigid (rotation and translation only) alignment is derived for selected regions of interest (ROIs) within bones. This ensures that bone rigidity is preserved in the final alignment.

In the work presented here, we applied the framework of Dzyubachyk et al. [7] to comparative visualization of MR images of the spine in patients with suspected axSpA. The aim of our study was to assess the feasibility of CA evaluation of axSpA inflammatory changes in the spine. This included fusion of scans from two time points into a single color-encoded image vividly distinguishing areas of increase versus decrease in inflammation over time, automatic labeling of VUs, and an interactive scoring module whose entry fields are activated/deactivated in synchronization with the VU selected by the reader in the image.

## Methods

### Patients

A total of 30 patients from the SPondyloArthitis Caught Early (SPACE) cohort were included in this feasibility study. The SPACE cohort has been described extensively before [8]. In short, the SPACE cohort is an ongoing cohort started in January 2009, including patients aged 16 years and older with chronic back pain (≥ 3 months, ≤ 2 years, onset < 45 years). All patients underwent a diagnostic work-up at baseline, consisting of history taking, physical examination, laboratory tests, and imaging (MR imaging (MRI) and plain radiographs). Patients fulfilling the Assessment of SpondyloArthritis (ASAS) axSpA criteria [9, 10] and patients with possible axSpA were included for follow-up visits after 3 and 12 months (including MRI). Possible axSpA was defined as the presence of at least one specific SpA-feature with a high positive likelihood ratio (LR+ above 6) or at least two less specific SpA-features (LR+ below 6), but not fulfilling the ASAS axSpA criteria [10, 11].

### MRI sequences

Patients underwent MRI of the complete spine in two stages (upper and lower spine) on a 1.5T MR system (Philips Medical Systems, The Netherlands). The acquired sequences were Short Tau Inversion Recovery (STIR) with repetition time (TR) 2500 msec, echo time (TE) 60 msec, inversion time 165 msec, acquisition matrix 304×300, echo train length (ETL) 25, number of averages 3 and T1-weighted Turbo Spin-Echo (TR 550/TE 10, acquisition matrix 512×305, ETL 5, number of averages 3). Imaging was performed in the sagittal plane with a field of view of 380×380 mm, slice thickness of 4 mm, and a slice gap of 0.4 mm.

### Vertebrae localization/segmentation/labeling

For each patient, 23 VUs were automatically localized, segmented, and labeled. A VU is defined as the region between the mid-points of two adjacent vertebral bodies. For example, VU1 consists of the lower endplate of vertebra C2 and the upper endplate of vertebra C3. Hence, VU levels 1–23 cover 24 vertebral bodies (C2–S1). Localization and segmentation were carried out using atlas-based segmentation [12]. The atlas set consisted of 11 patients from the SPACE cohort (no overlap with patients included in evaluation). For each atlas patient, 24 vertebral bodies (C2 to S1) were manually outlined in the slice closest to the mid-sagittal plane and the two adjacent slices (a total of three slices). The procedure was carried out separately for upper and lower spine images, producing a total of two manually segmented images per atlas patient. We chose to approximate each vertebral body with a simple polygonal region within the vertebral borders, taking the cortex as an anatomical boundary. This choice was motivated by the fact that for successful locally rigid alignment of two time points it is preferable to have a ROI estimate that under-segments the bone, rather than an estimate that spills over into inherently non-rigid neighboring soft tissue.

The first phase of atlas-based segmentation consisted of image registration between each of the 11 atlas patients and the target patient being segmented. Image registration was performed using the Elastix software package [13, 14]. After spatially mapping vertebrae ROIs from every atlas image onto the target image, a majority vote was applied across all mappings to determine whether a voxel was part of the background or of one of the vertebrae.

Labeling of vertebrae voxels in the upper spine image was done sequentially from top to bottom, over connected components, with the top-most connected component receiving the label “C2,” the following “C3,” etc. Similarly, labeling in the lower spine image was done sequentially from bottom to top, with the bottom-most connected component receiving the label “S1,” the following “L5,” etc. We used a 26-connected neighborhood definition for connectivity in 3D. Connected components less than 20 voxels in size were considered to be noise and were removed.

### Locally rigid inter-time point alignment

In what follows, let us consider a pair of MR scans of a single patient and, without loss of generality, refer to one of the scans as “Time Point 1 (TP1)” and the second scan as “Time Point 2 (TP2).” According to the framework proposed by Dzyubachyk et al. [7], locally rigid alignment of two images is derived from a global non-rigid alignment of this image pair. We used the Elastix software package [13, 14] to globally align TP2 to TP1. The registration yielded a deformation field specifying for each physical position in TP1 the corresponding physical position in TP2. Next, for each VU, the landmark transform [15] was used to estimate a locally rigid alignment between the VU region in TP1 (specified by the atlas-based segmentation result) and the corresponding physical region in TP2 (specified by the deformation field) [7]. This ensured that spatial correspondence between voxels within the VU in TP1 and TP2 was restricted to translation and rotation, preserving bone rigidity.

It is important to note that the described method can be equivalently applied in the reverse direction, by globally aligning TP1 to TP2, and subsequently using VU segmentations from TP2. Thus, in order to align two scans in a locally rigid manner, it is sufficient to segment and label vertebrae in one of the two scans.

### Color-encoded fusion of time points

**Fig. 1:**
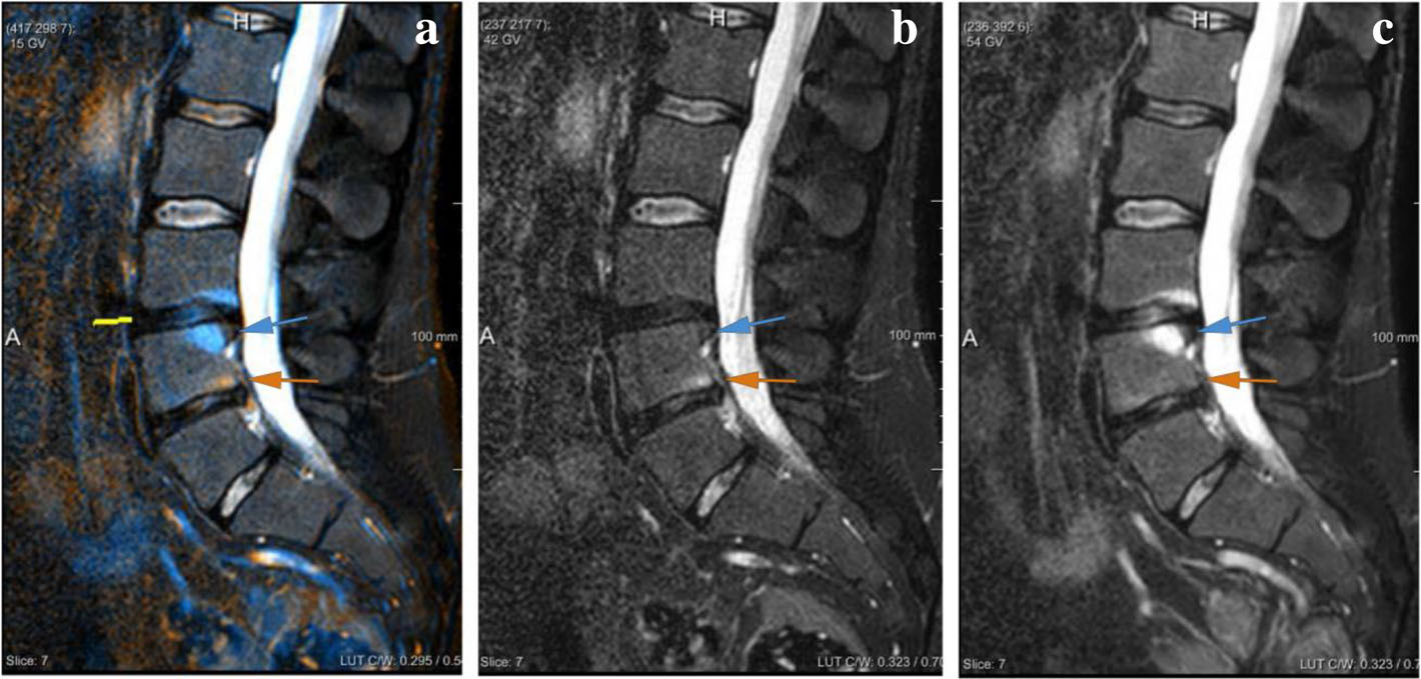
Color-encoded fusion of two MR scans of the same subject acquired at two different time points. Inflammation increase (blue arrow) in VU21 and decrease (orange arrow) in VU22 in the second time point (**c**) compared to first time point (**b**) are displayed in blue and orange, respectively, in the color-encoded fusion image (**a**). In this example, the locally rigid alignment is applied to VU21, indicated by the yellow line in (**a**)

After locally aligning two time points on the VU level, differences in intensity (e.g. inflammation) between corresponding voxels were visualized through color-encoded fusion of the two scans. First, intensity values of TP1 were color-mapped to orange color space (RGB triple {255,128,0}), and intensity values of TP2 were color-mapped to light blue color space (RGB triple {0,127,255}). The fusion image was then obtained by voxel-wise superposition of the two color-mapped images [7]. Since orange and light blue are complementary colors, areas where no changes occurred between the two time points (TP2 intensity = TP1 intensity) are displayed in shades of gray. On the other hand, an increase in inflammation over time (TP2 intensity > TP1 intensity) is displayed in shades of light blue (Fig. 1). In the opposite case, a decrease in inflammation over time (TP2 intensity < TP1 intensity) is displayed in shades of orange. In addition to its complementary nature, the choice of orange and light blue is motivated by the fact that these two colors can be perceived even by readers with color vision deficiency [7]. No intensity standardization was applied to original images prior to color-encoded fusion.

### Evaluation of inflammatory changes

**Fig. 2:**
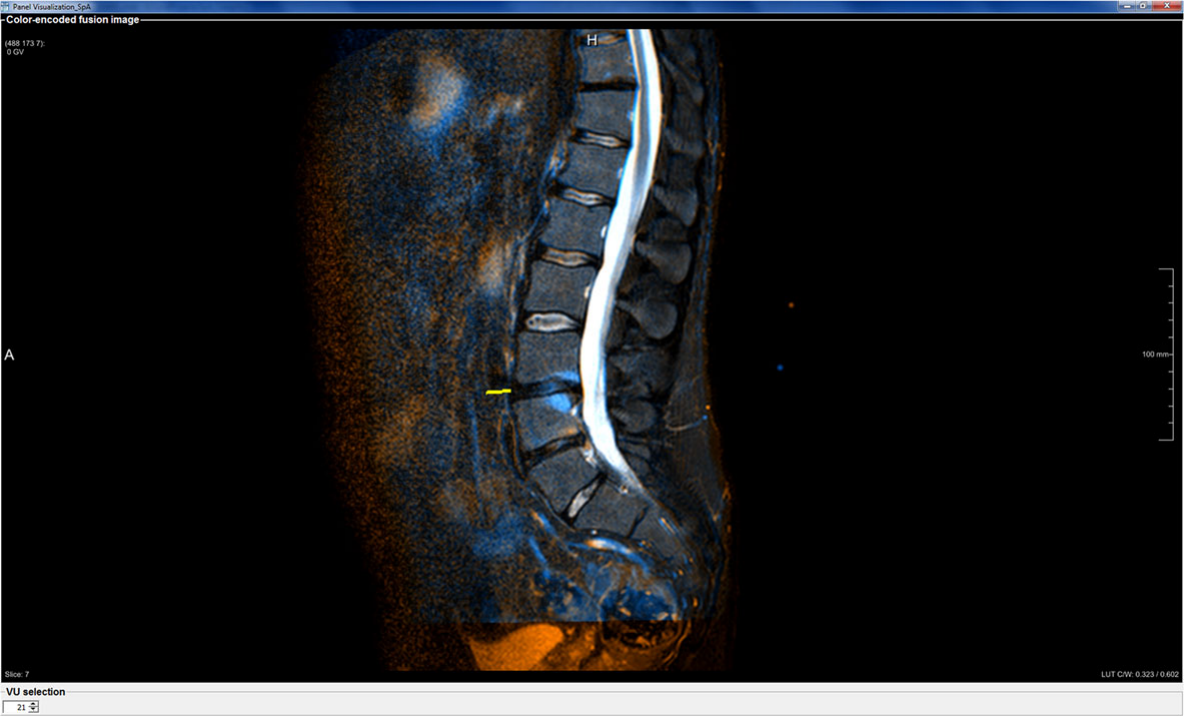
Comparative visualization module. The module displays the color-encoded fusion image and allows the user to specify the VU of interest in the VU selection field at the bottom left of the window, which will trigger locally rigid alignment of two time points for that VU. A visual indication for the position of the VU in the image is provided to aid navigation (yellow line next to VU 21)

**Fig. 3:**
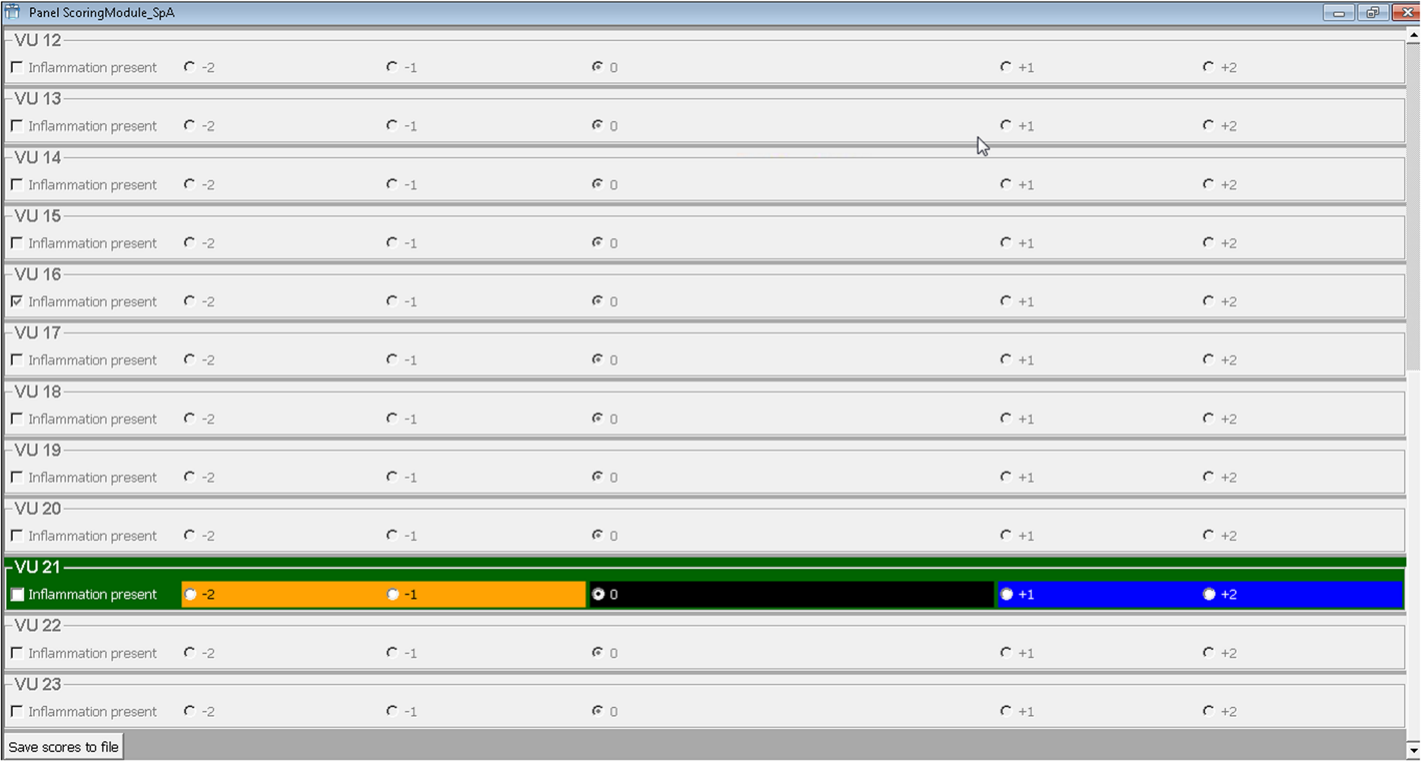
Scoring module. The module acts as an interactive scoring sheet, consisting of 23 panels representing the VUs. Every panel contains a group of option buttons (only one of the options can be selected) through which the reader assigns a change score to the VU, as well as a checkbox to indicate the presence of inflammation in cases of no net change. Only one VU panel is active at any given moment. The choice of VU in the comparative visualization module activates the corresponding panel in the scoring module, while deactivating panels of the remaining 22 VUs. This ensures that one VU is not mistaken for another while filling out the interactive scoring sheet

Two experienced readers (RvdB and ZEZ) independently evaluated inflammatory changes between MR scans of the spine (STIR only), acquired 9–12 months apart, directly from the color-encoded fusion image. The choice of using only STIR images for CA scoring was motivated by our focus on inflammatory lesions and the fact that automatic alignment of T1 images to STIR images requires additional image registration steps, which would introduce additional sources of error. The readers were blinded to the original images and their time order, as well as patient and clinical characteristics. Each VU was assigned a score ranging from −2 (dramatic decrease of inflammation), via 0 (no change), to +2 (dramatic increase of inflammation), reflecting net change in the degree of inflammation within the VU. Navigation through the images and evaluation were carried out using an interactive software tool that we implemented in MeVisLab 2.7.1 (MeVis Medical Solutions, Germany) [16]. The tool consists of two windows: the comparative visualization module (Fig. 2) and the scoring module (Fig. 3).

For comparison of direction of change (increase/decrease) indicated by an existing reference, Berlin method [1] scores of the same pair of MR scans from a different ongoing SPACE cohort study at our institution were used. The MR scans (STIR and T1) were independently evaluated by two experts (MdH and PACB) according to the Berlin method [1], yielding status scores for each of the time points. Each VU was assigned a score ranging from 0 to 3 reflecting the fraction of bone volume affected by bone marrow edema: 0, normal; 1, < 25% VU edematous; 2, 25–50%; 3, > 50%. The readers were blinded to the time order of the images, as well as patient and clinical characteristics. Changes in inflammation over time were calculated as differences in status scores after de-blinding the time order of the MR scans.

### Statistical analysis

For each of the 23 VU levels, the distributions of VU-level inter-reader and inter-method differences across patients were analyzed descriptively. For inter-reader differences, the VU-level difference was computed between change scores assigned to the VU by the two CA readers. For inter-method differences, the focus was on the direction of change indicated by each method, and therefore, VU-level difference was computed between the sign of the CA change score (mean of two readers) and the sign of the Berlin change score (mean of two readers), where the sign function takes the value −1 in case of negative change, +1 in case of positive change, and 0 in case of no change.

Agreement between CA readers was assessed on the patient level (change summed across VUs of each patient) by computing intraclass correlation coefficient (ICC, two-way mixed, single measures, absolute agreement definition). The statistics were computed using MATLAB R2015b (The MathWorks, Inc., USA) and IBM SPSS Statistics 23 (IBM Corporation, USA).

## Results

In 18/30 patients, atlas-based segmentation provided satisfactory segmentation and correct labeling of all 23 VUs in at least one of the time points (as explained above, in order to align two scans in a locally rigid manner, it is sufficient to segment and label vertebrae in one of the two scans). In seven patients, failure to segment the lowest vertebra in the upper spine image and/or the highest vertebra in the lower spine image, resulted in lack of segmentation for one VU (frequently corresponding to the levels Th9–Th11). The segmentations for these VUs were added by manual correction. Five patients were excluded from further evaluation due to inaccurate alignment with atlas images that led to missing vertebrae segmentations and incorrect labeling of VUs. Thus, a total of 25 patients (and hence 575 VUs) were evaluated. Baseline patient characteristics and descriptive statistics of Berlin and CA scores at baseline and over time are presented in Table 1. As demonstrated by baseline characteristics, it should be pointed out that most patients had low levels of inflammation.

**Table 1:** Baseline patient characteristics and descriptive statistics of Berlin and CA scores of the 25 patients evaluated in the study

Baseline patient characteristics		
Characteristic	Patients (*n* = 25)	
Age at inclusion in years, mean (SD)	31.7 (8.3)	
Male, *n* (%)	12 (48)	
Duration of back pain in months, mean (SD)	14.4 (8.0)	
BP, *n* (%)	19 (76)	
HLA-B27 positivity, *n* (%)	15 (60)	
Elevated CRP, *n* (%)	6 (24)	
Sacroiliitis on MRI (ASAS definition), *n* (%)	8 (32)	
Sacroiliitis on radiograph, *n* (%)	3 (12)	
Positive MRI (ASAS definition), *n* (%)	2 (8)	
ASAS classification positive, *n* (%)	14 (56)	
Berlin and CA scores descriptive statistics		
Variable	Berlin method (reader 1/*reader 2*)	CA method (reader 1/*reader 2*)
VU-level score at baseline, median (range)	0 (0, 1) / *0 (0, 1)*	NA
VU-level score at follow-up, median (range)	0 (0, 1) / *0 (0, 1)*	NA
Patient-level score at baseline, median (range)	0 (0, 5) / *0 (0, 3)*	NA
Patient-level score at follow-up, median (range)	1 (0, 5) / *0 (0, 4)*	NA
Change in VU-level score, median (range)	0 (−1, 1) / *0 (−1, 1)*	0 (−1, 2) / *0 (−2, 2)*
Change in patient-level score, median (range)	0 (−2, 3) / *0 (−1, 2)*	0 (−3, 3) / *0 (−11, 5)*

### Inter-reader differences between CA readers

**Fig. 4:**
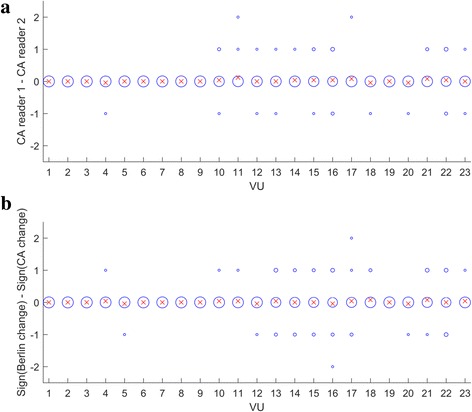
VU-level difference between CA readers’ change scores (**a**) and VU-level difference between sign of CA and Berlin change scores (**b**). Exact difference values are shown in blue (size of bubble data points is proportional to the occurrence of the difference value across 25 patients). Mean VU-level differences across 25 patients are shown in red

VU-level differences between CA readers’ change scores are shown in Fig. 4a. Mean VU-level differences across patients ranged from −0.04 to 0.12 with standard deviation (SD) range (0, 0.45). Most differences were observed in the lower thoracic spine and the lumbar spine. In 21/23 VU levels, differences ranged between −1 and 1 across all patients. In 2/23 VU levels, a difference of 2 was observed in one patient. In total, across all patients, VU-level differences ranged (−1, 1) in 573/575 VUs (99.7%). On the patient level, the ICC between the two CA readers was 0.56 (95% confidence interval (CI): 0.22 to 0.78), indicating moderate agreement between readers.

### Inter-method differences between CA and Berlin methods

**Fig. 5:**
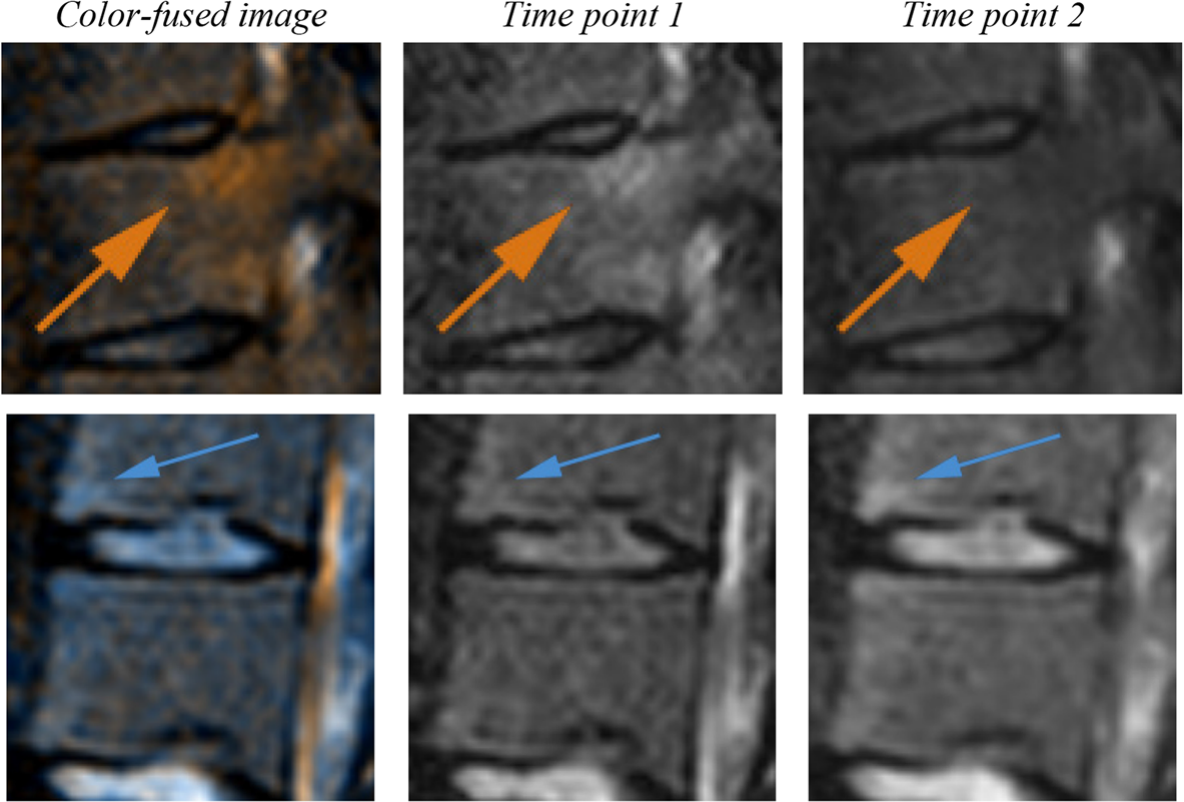
Examples of VU-level inter-method differences. Top row: lesion area (orange arrow) received a CA change score of −1, but a Berlin change score of 0, because of being considered a degenerative lesion (status scores = 0). Bottom row: lesion area (blue arrow) received a CA change score of 1, but a Berlin change score of 0, because of zero Berlin status scores

VU-level differences between the direction of change indicated by the CA and Berlin methods are shown in Fig. 4b. Mean VU-level differences across patients ranged from −0.04 to 0.08 with SD range (0, 0.61). Most differences were observed in the lower thoracic spine and the lumbar spine. In 21/23 VU levels, differences ranged between −1 and 1 across all patients. In 1/23 VU levels, a difference of 2 (positive Berlin change, negative CA change) was observed in one patient. In 1/23 VU levels, a difference of −2 (negative Berlin change, positive CA change) was observed in one patient. In total, across all patients, VU-level differences ranged (−1, 1) in 572/575 VUs (99.5%). Differences of precisely −1 or 1 (change detected only by one of the two methods) were observed in 40/575 VUs, and among those in 33/40 VUs the change was detected by the CA method while Berlin score indicated zero change. Figure 5 shows examples of VU-level differences between the two methods.

## Discussion

In this study, we assessed the feasibility of CA evaluation of inflammatory changes on MR scans of the spine in patients with suspected axSpA. Readers agreed that a key advantage of CA evaluation is that fusion of two scans acquired at different time points into a single color-encoded image allows for direct visualization and measurement of inflammatory changes, as opposed to derivation of change scores from status scores that measure presence and extent of lesions separately for each time point. The results indicate that in nearly all VUs of all patients, VU-level differences between CA readers and between the CA and Berlin methods were bounded between −1 and 1, ensuring that scores do not offer opposing opinions on the direction of inflammatory change (increase versus decrease). The fact that most differences occurred in the lower thoracic spine and the lumbar spine is consistent with the observation that most inflammatory activity in the spine of early disease patients takes place in these regions [17, 18]. The majority of non-zero differences between the CA and Berlin scores were observed when change was detected by the CA method while zero change was indicated by the Berlin method. These quantitative results support our qualitative observation that small gradual changes in an existing lesion are often not reflected in Berlin status scores, but can be readily visualized and measured by the CA method.

The moderate inter-reader patient-level agreement and difference in the range of readers’ scores suggest that the CA grading scale may be defined too loosely with respect to affected bone volume, making the score more susceptible to subjective interpretation of the degree of change. Readers pointed out that a challenging aspect of the CA method is estimation of net inflammatory change in VUs with multiple inflammatory lesions. For example, one such VU exhibited increase in one quadrant, while exhibiting decrease in another quadrant. The two readers had different opinions as to which change was stronger, resulting in opposing scores and thus a mean change of zero. One way to overcome such discrepancies would be to score change separately for each of the four quadrants, akin to scoring in the Spondyloarthritis Research Consortium of Canada (SPARCC) method [2].

For the purpose of this feasibility study, we made a deliberate decision to measure change based only on the color-fused image, while blinding readers to the original images. However, the readers noted that in daily practice it would be helpful to have the original images (STIR and T1) available next to the color-fused image, as this would contribute to a more informative scoring decision. The color-fused image could then be used as a map that directs the reader’s attention to locations of potential inflammatory changes, while the original images would be used to make the final judgement about the type and degree of observed change. The reader would still benefit from locally rigid alignment between the two time points while assessing original images, since the two scans will be aligned such that the VU of interest has identical viewing points in both images. Another feature that would enhance user experience is stitching of upper and lower spine images into a single image. This would offer a more natural workflow, without the need to load two separate images for every patient. A simultaneous view of the complete spine would also facilitate a more holistic assessment of disease activity.

This study has several important limitations. The SPACE cohort consists only of early disease patients with low levels of inflammation, making it harder to study inflammatory changes, since changes were infrequent. Another limitation is that it was not possible to provide patient-level inter-method agreement statistics, as the scoring methodology and scale range are different, and this would result in uninterpretable results. However, the CA method was not designed with the aim of replicating the Berlin method, but rather as an independent scoring framework. It is of interest to compare the responsiveness of the two methods by quantifying sensitivity to change in a population with treatment and placebo patient groups, as was previously done for other scoring methods [19]. Assessing responsiveness after an effective intervention could provide information on differences in the psychometric characteristics of the two scoring methods. This could be addressed in a follow-up study. The definition of the CA change score should also provide clear guidelines for the case of degenerative lesions to avoid discrepancies with existing methods that do not score these lesions (Fig. 5). The lack of intensity standardization prior to color-encoded fusion is a potential source of error. However, we should note that standardization is also not applied in the long-established procedure of Berlin method scoring. We sought to explore the feasibility of color-encoded fusion without additional image post-processing steps that are not present in the Berlin method workflow. Future studies should indeed investigate the effect of intensity standardization on change scores of both methods. An additional limitation is that CA scoring was performed only using STIR images. This differs from the common clinical approach of confirming inflammatory lesions observed in STIR images as low intensity areas in T1 images. Inclusion of T1 images may improve the robustness of the scoring method. Furthermore, it might allow visualization of changes in structural lesions, such as fatty lesions. Finally, it should be recognized that since 5/30 patients had to be excluded due to failed segmentation and 7/30 segmentations had to be manually adjusted, the method is not yet sufficiently robust to be used in practice. We should stress, however, that this study did not attempt to solve the problem of vertebrae segmentation in MRI. Our goal was to explore the prospect of CA assessment of patients with suspected axSpA and thereby provide yet another stimulus for development of robust vertebrae segmentation methods for MRI.

Although this study does not focus on the topic of vertebrae segmentation, we can note potential directions for improving the atlas-based segmentation framework used in this study. To ensure the applicability of this segmentation framework to a variety of MRI acquisition protocols and scanners, it would be helpful to construct an atlas consisting of sub-atlases of MR images acquired under similar echo/repetition times and magnetic field strengths. Then, prior to segmenting a target image, the system would automatically identify the most appropriate sub-atlas based on acquisition parameters recorded in the image’s DICOM data. Additional improvement in segmentation might be achieved by operating with stitched images of the spine, as opposed to separate upper/lower spine images. We have observed that in some cases segmentation was successful in one part of the upper/lower pair but failed in the other. Therefore, we hypothesize that the more easily matched spine region can “pull” the second spine region into its correct position in the target image when stitched.

## Conclusions

This feasibility study has demonstrated that fusion of MR scans of the spine from two time points into a single color-encoded image allows for direct visualization and measurement of inflammatory changes over time in patients with suspected axSpA. A future study, with similar design to that of Lukas et al. [19], should assess the performance of the CA method in patients with a wide range of activity at baseline and follow-up, quantifying inter-reader reliability, sensitivity to change, and time needed to score each set of MR images. This would also provide a comprehensive comparison of the CA method to the Berlin and SPARCC methods.

## Abbreviations

ASAS: Assessment of SpondyloArthritis
axSpA: axial SpondyloArthritis
CA: Computer-aided
CI: Confidence interval
DICOM: Digital Imaging and Communications in Medicine
MR: Magnetic resonance
MRI: Magnetic resonance imaging
RGB: Red, Green, Blue
ROI: Region of interest
SD: Standard deviation
SPACE: SPondyloArthitis Caught Early
SPARCC: Spondyloarthritis Research Consortium of Canada
STIR: Short Tau Inversion Recovery
TE: Echo time
TP1: Time Point 1
TP2: Time Point 2
TR: Repetition time
VU: Vertebral unit
